# Abstract of the Proceedings of the Buffalo Medical Association, June, 1874

**Published:** 1874-07

**Authors:** Joseph Fowler

**Affiliations:** Secretary, pro tem.


ART. IV.—*Abstract of Proceedings of Buffalo Medical Association*, June 2d, 1874.

Reported by Joseph Fowler, M. D. Secretary, *pro tem.*

The Association met at the usual time and place. Members present, Drs. Rochester, Wyckoff, Johnson, Brecht Shaw, Bailey, Walsh, Fowler.

In the absence of the permanent president and secretary, on motion of Dr. Rochester, Dr. Shaw was called to the chair, and Dr. Fowler made temporary secretary.

Dr. Wyckoff moved that Dr. Boysen be invited to participate in the proceedings of the meeting. Adopted. On motion of Dr. Rochester the same invitation was extended to Dr. W. W. Miner.

Dr. Rochester, chairman of special committee appointed at the last regular meeting, to settle with the Real Estate Commissioners of the Young Men's Association, reported that he has paid to them the amount of one hundred dollars, this sum being the entire indebtedness of this association to them, and presented a receipt for the same.

The report was received and committee discharged.

Reports of prevailing diseases being in order, Dr. Wyckoff said, that owing to his absence from the city, he could not form a very definite opinion concerning those diseases which prevailed most extensively, but since his return had seen some scarlatina, and had two fatal cases.

One case, a girl aged six years, died Sunday having been ill about a week. The eruption could never be distinctly seen. Brain symptoms were developed early, slight convulsions being present. In a few days the intellect became clear, and convalescence quite fairly established, when suddenly diphtheritic complication developed itself, the fauces being copiously covered with the characteristic exudation.

A majority of the other cases presented decided brain symptoms. Would like to know if scarlet fever assumes that type.

Dr. Rochester said he reported at the last meeting the existence of but few cases, and was hopeful that the prevalence of the disease was rapidly declining, but was sorry to say that his anticipation was not realized.

Since that time he had seen six fatal cases, two having the diphtheritic complication as mentioned by Dr. Wyckoff.

Two of these cases had occurred in his private practice, and the others he had seen in consultation.

One on Park street was well Thursday at 1½ P. M., was called to see it at six P. M., of the same day, found feeble pulse, livid skin, coldness of extremities, little attempt at eruption, and constant emesis. Patient died on the following day.

Another in the same family, aged three years, was taken at 9 P. M., with similar symptoms and died the next morning.

A third child in the same family was sent from home, that it might escape the disease, and has done so.

Saw another with Dr. White on Delaware street. The patient died the day following.

Saw still another with Dr. Mixer,—a girl aged 6 years. This patient did not appear to be very sick on the fourth day. On the fifth day the eruption disappeared—the patient dying two days later with diphtheria.

These are exceptional cases, the majority having been of a mild character during the past winter, the severe cases having appeared later.

Dr. Boysen reported a case of scarlet fever in a person 23 years of age, with marked brain symptoms present, and the absence of any eruption. The patient died on the fifteenth day from the attack.

Dr. Wyckoff desired to relate another case that he had watched with much interest.

It was in the person of a lady 35 years old, quite feeble, and in delicate health. She had slept with her child while it had scarlatina, which was of a mild character, terminating favorably, without any serious complications.

The mother contracted the disease and presented all the constitutional symptoms without the eruption.

On the fifth day from the attack diphtheritic deposit was observed in the fauces extending to the trachea, producing entire loss of voice accompanied with dyspnœa. She has since entirely recovered from the disease, but is unable to speak.

All members present concurred in the continued prevalence of Parotitis, Pertussis, and Typho-Malarial Fever.

Voluntary Communications being in order, Dr. Wyckoff, said he was called to see a gentleman aged 63, who complained of violent pain in the right side, which was relieved with full doses of morphine administered subcutaneously, and repeated every three or four hours. Subsequently the pain was more marked following the course of the ureter on the same side.

On the second day peritoneal inflammation was developed accompanied with pain and retraction of the right testicle.

The peritonitis became general, accompanied with great tenderness, pain and tympanitis. Patient died on the fourth day from the attack.

Dr. Rochester wished to relate a case that properly came within the domain of surgery, although making no claims as an expert in that department.

A young woman called to see me, stating that she had run a needle into her hand while washing curtains. There was pain running up the arm, and general *malaise*. On examination I thought I could feel something and made an incision; spent half an hour searching for it, but failed to find it. Sent her home and ordered a poultice to the part.

In a few days my patient returned with all the symptoms very much aggravated. I extended the incision and finally succeeded in removing the fragment. She manifested some symptoms of tetanus.

I mention this case since it is the general practice to make no attempt to remove needles, if they can not be distinctly felt or seen, and that they are sometimes found.

Dr. Bailey reported a similar case where he had cut down and failed to discover the portion of a needle that was known to be there. After applying a poultice for a few days, he succeeded in detecting it, but was only able to remove it by bringing into requisition the use of a strong pair of shoemaker's pincers, and the employment of much physical strength.

Dr. Shaw read the following paper, after which he wished the opinion of those present as to the cause of the retention of the cherry stones in the alimentary canal.

The first day of April, 1874, was called to 175 Pratt street, to see a child nearly six years old, the daughter of young, strong and healthy German parents, there being two other children younger, both healthy.

This child was, and had been for several days, suffering frequent paroxysms of severe pain in the abdomen, which was largely distended, and accompanied with firm resistance at each paroxysm of pain at all parts of the abdomen, but more especially at the umbilicus, not entirely unlike labor pains, and fearful contortion of the countenance at each paroxysm. These paroxysms occured at times as often as once in three or five minutes, and were frequently accompanied with nausea, and occasionally with vomiting.

This condition of things was only partially relieved by the best evacuation that could be obtained from the use of castor oil as a cathartic, which evacuations were always obtained with more or less difficulty, as represented by the mother.

This resort to the use of oil was observed by the mother to be necessary immediately after the stoppage of the rattling in the belly, as she termed it, or the borborygmus, which was a prominent and almost a constant symptom. The child looked pale and haggard, but had not that peculiar cadaverous look that is ever present in Tabes Mesenterica, or consumption of the bowels; nor that peculiar uneven, lumpy, corrugated condition of the abdominal viscera, so readily found or made manifest through the parietes of the abdomen, in that fearful disease.

This child had been under treatment, at short intervals, for over two years and a half, and had well nigh exhausted all schools of medicine, with no permanent relief. The pain and bloated condition would partially subside, but only for a short time, then they would return again with seemingly increased torture and deformity.

From the best information that I could obtain of the plan of treatment pursued by my predecessors in the case, (which were some of the prominent practitioners of this city), it was the cod-liver oil treatment, which was to me pretty good evidence that Tabes Mesenterica was suspected to exist.

Yet, after all the evidence, I could see no other plan for me to pursue but to open the bowels thoroughly, if it could be done. To effect such a purpose I ordered Pillula Hydr. and Sub. Mur. Hydr. of each xv grains, well incorporated into three pills, to be given at once, and followed in five hours with castor oil, if required to open the bowels. Then I ordered, if requisite to open the bowels, to administer by the Rectum as large a quantity of tepid water as could be used, two quarts if practicable, but no water was used.

The bowels moved as of old from the effects of a full dose of calomel, but the evacuations started reluctantly and continued to hesitate and hold back. Yet, within 24 hours, a large quantity of fecal matter passed away, with forty or fifty cherry pits, as stated by the parents, twenty of which I have in my possession, that appear to have been made smooth by trituration, and are as black as a crow.

These pits must have lain in the bowels about two years and eight months, as stated by the parents, as the child got to a basket of cherries and helped herself, and ate largely of them, and swallowed pits and all; the child also ate freely of cherries all through the season in the same manner. The child was taken sick the October following.

After this thorough clearing out of the bowels the bloat subsided and the rattling ceased without complaint from the child for the first time in over two years.

And now, after five weeks time, the bloat has not returned to any great extent, nor the borborygmus. The child has a voracious appetite, yet has some pains in the bowels at times, and some bloating.

Now the question arises: Were the cherry stones retained by a diseased or contracted condition of the intestinal canal? Or, were the cherry stones retained in a normal condition of the bowels the cause of the whole disturbance? Would a full dose of sub muriate have removed the obstruction at the time it first occurred? Inasmuch as insufficient time has elapsed to give the recuperative powers of the system an opportunity to restore and regulate lost functions, and as the child continues to complain of pain in the bowels at times, I will leave the case open for further thought and consideration, and further report in future.

June 2nd, 1874. After two monthe have elapsed I am able to say that I have left the case mostly to the Vis Medicatrix Naturæ, and can report the little patient restored to a normal condition of size, shape, health and comfort, with an occasional bloat and rattling in the belly for a short time, and then it all passes away.

Dr. Rochester thought they had been impacted in the intestines as their smooth surfaces would indicate.

Under the head of miscellaneous business, Dr. Walsh said he had received intelligence of the death of Doctor Burke, at Riverside, California.

Since he had been a member of this Association, he moved that the chair appoint a committee of three to draft appropriate resolutions. Adopted.

The chair appointed as such committee, Drs. Rochester, Johnson and Walsh.

On motion of Dr. Wyckoff the Association adjourned.

